# Ectopic extramammary Paget’s disease initially detected by lymph node metastasis^؆^

**DOI:** 10.1016/j.abd.2026.501475

**Published:** 2026-11-04

**Authors:** Tatsuhiko Mori, Takehiro Nakamura, Riko Ishizaki, Kinuko Irie, Takashi Ito, Toshiyuki Yamamoto

**Affiliations:** Department of Dermatology, School of Medicine, Fukushima Medical University, Fukushima, Japan

Dear Editor,

A 61‑year‑old Japanese woman with a history of hypertension and dyslipidemia presented with progressive right inguinal lymphadenopathy. Computed tomography demonstrated multiple enlarged lymph nodes in the inguinal, pelvic, and para‑aortic regions (Fig. 1a‒b). Fine‑needle biopsy performed at another institution revealed adenocarcinoma. Despite extensive evaluation ‒ including positron emission tomography-computed tomography, gastrointestinal endoscopy, breast and gynecological examinations ‒ the primary tumor could not be identified, and the patient was referred to our oncology service as carcinoma of unknown primary. On dermatologic consultation, we noted a chronic erythematous scaly plaque mimicking Bowen’s disease on the right lower abdomen, which was present for more than 10-years but left unexamined (Fig. 1c‒d). Skin biopsy of the abdominal lesion revealed numerous atypical cells with pale cytoplasm infiltrating predominantly within the epidermis and focally into the dermis. Immunohistochemistry was positive for AE1/AE3, EMA, CK7, CAM5.2, and CEA, focal positivity for GCDFP-15 and CK20 (Fig. 2a–d). The right inguinal lymph node biopsy showed atypical cells with eosinophilic cytoplasm. Immunohistochemistry was positive for AE1/AE3, EMA, CK7, CAM5.2, CEA, and HER-2(2+), while negative for GCDFP-15 and CK20 (Fig. 3a–d). These findings confirmed Ectopic Extramammary Paget’s Disease (E-EMPD) with lymph node metastases. The primary lesion had a tumor thickness of 0.3 mm without lymphovascular invasion. According to the TNM staging system proposed by the Japanese Dermatological Association and the Japanese Skin Cancer Society, E-EMPD of the patient was classified as T1N2M1, stage IV. The patient was started on nivolumab (400 mg/body weight every 4-weeks) as first-line treatment, which is ongoing after 12-months. The right inguinal nodes regressed; however, contralateral inguinal and pelvic nodes enlarged. Gene panel testing (GeniMine TOP, Konica Minolta, Inc.) identified several genomic alterations, including DNMT3A, EGFR, and PALB2 as well as a high tumor mutation burden (23.0 mutations per megabase). However, these findings did not indicate any actionable therapeutic options.

**Fig. 1:**
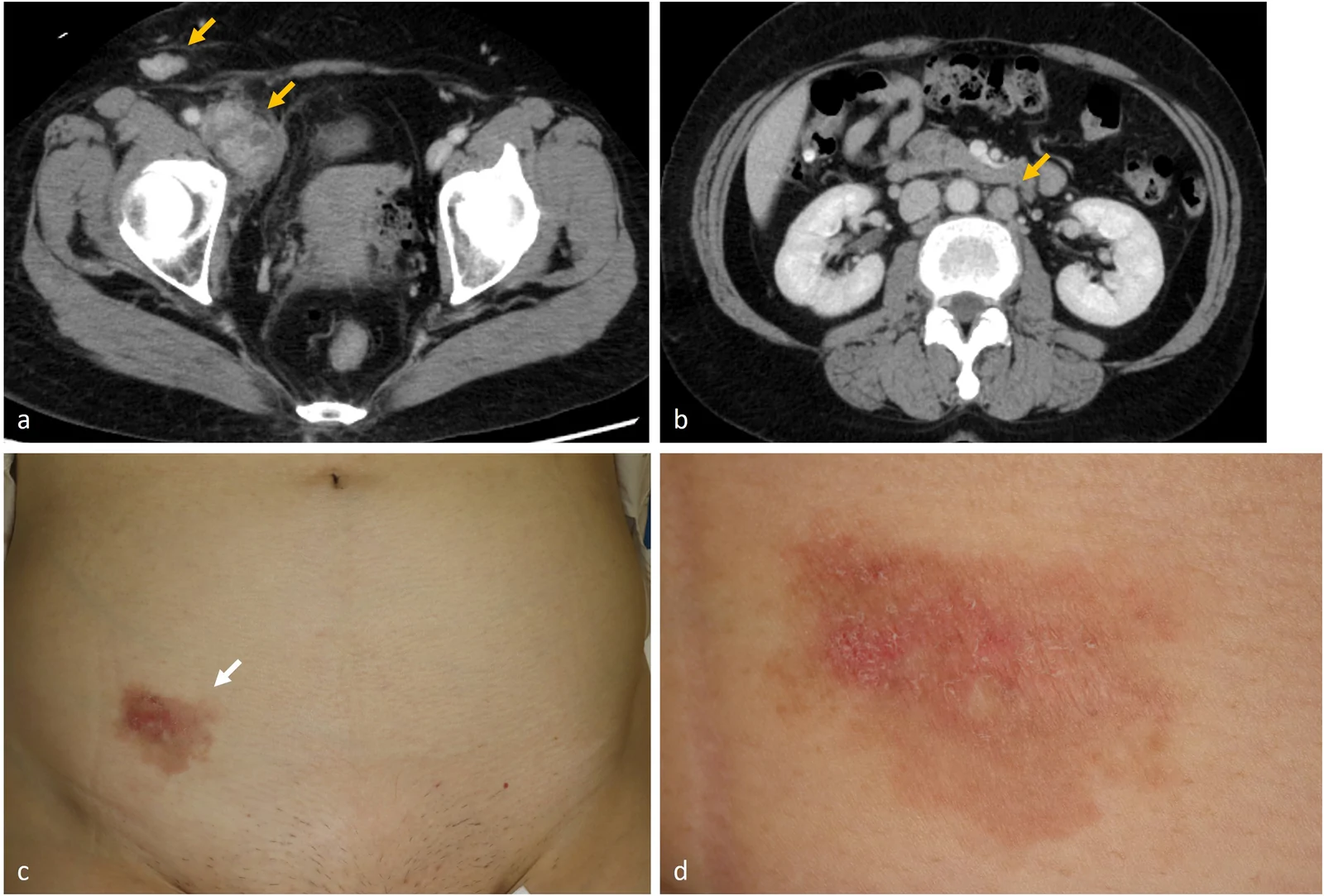
Clinical and radiological findings of the patient. (a–b) Computed tomography images showing enlarged right inguinal, right pelvic and para-aortic lymph nodes (arrow). (c–d) Scaly red-to-brown patches on the right lower abdomen (arrow).

**Fig. 2:**
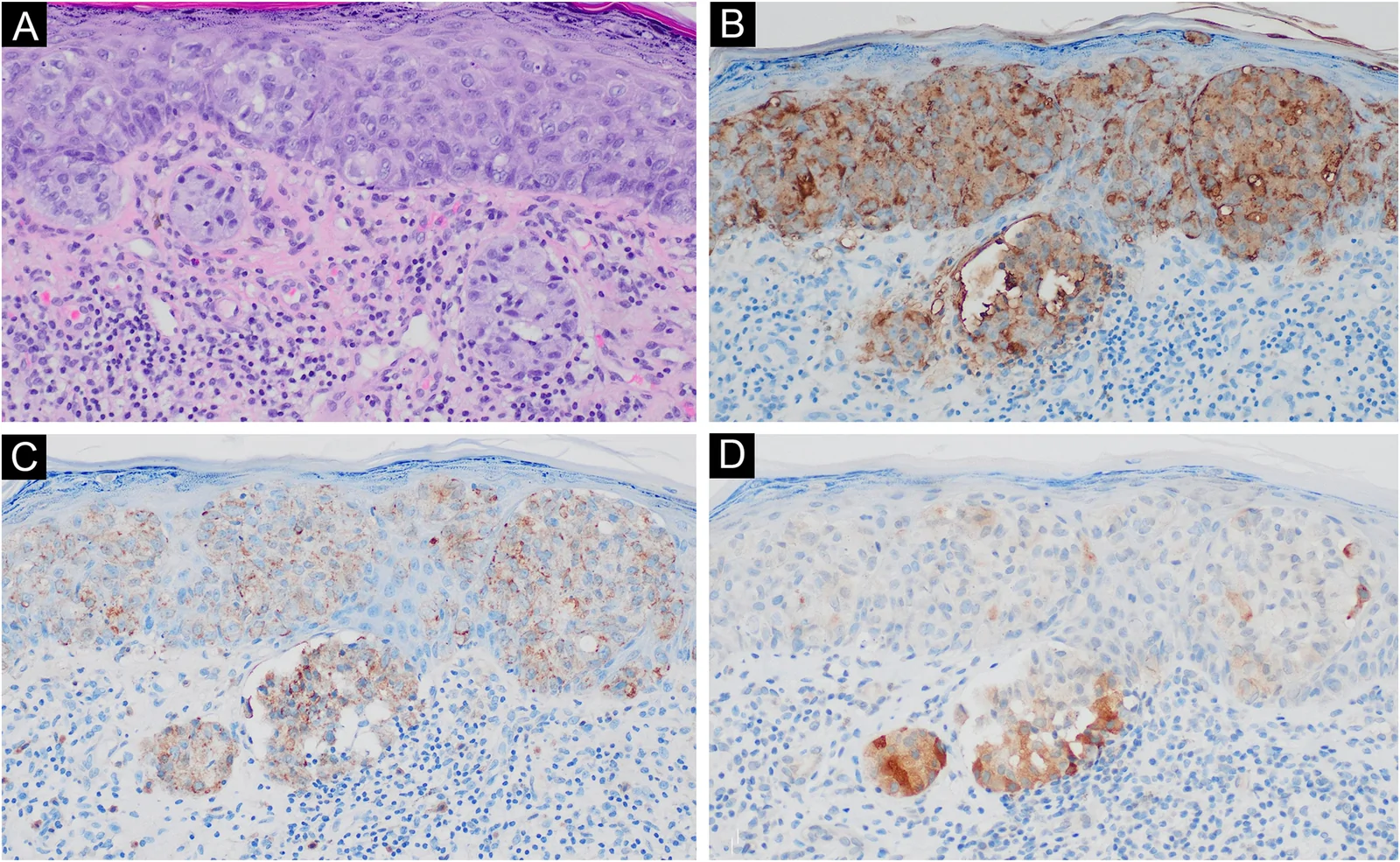
Histopathological examination of the skin lesion. Atypical Paget cells with pale cytoplasm within the epidermis and dermis were shown on Hematoxylin & eosin staining (a). Immunohistochemistry demonstrated positive staining for CEA (b), CAM5.2 (c), and HER2 (d). Original magnification ×200.

**Fig. 3:**
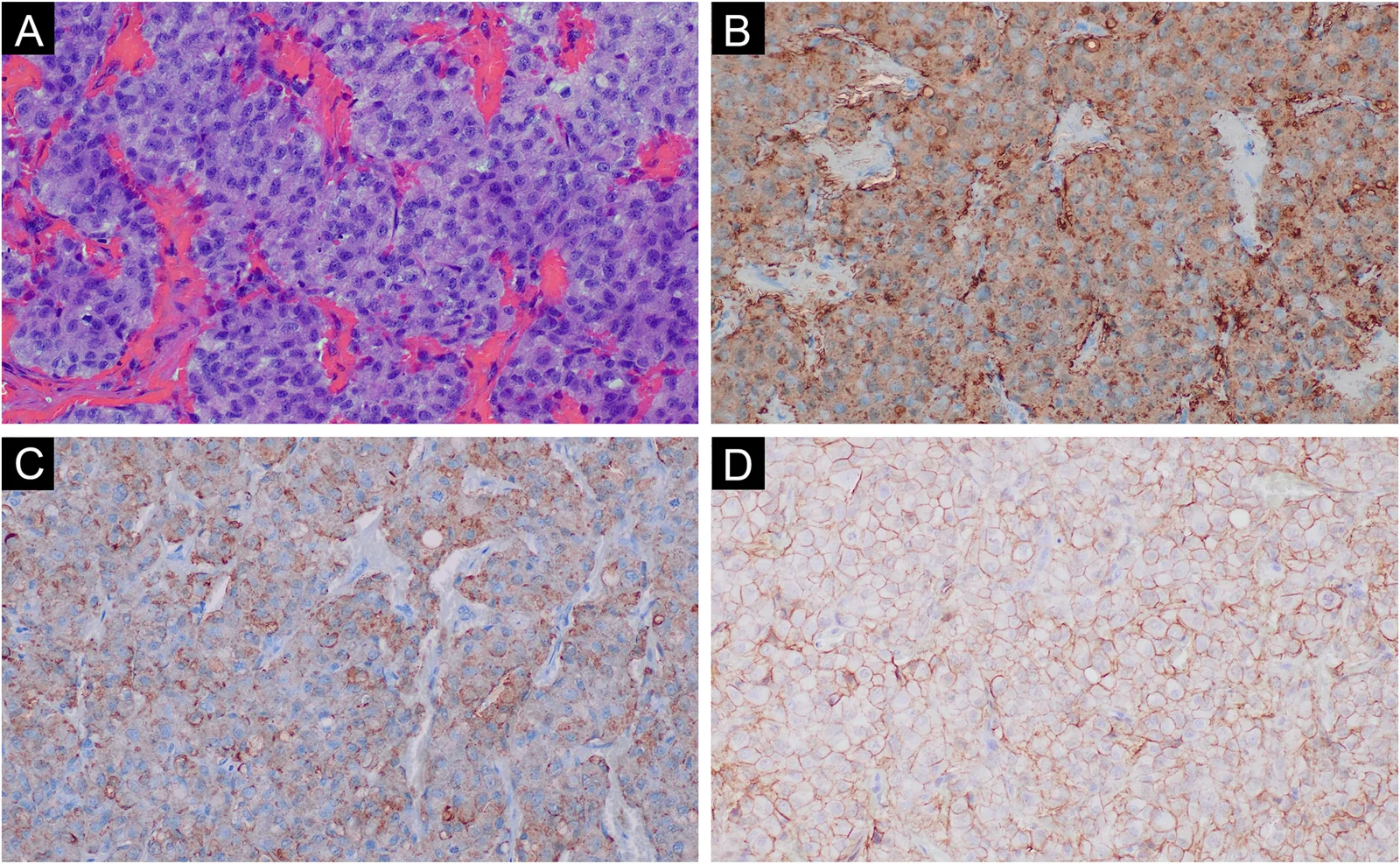
Histopathological examination of the inguinal lymph node. Atypical eosinophilic cells were shown on Hematoxylin & eosin staining (a). Immunohistochemistry demonstrated positive staining for CEA (b) and CAM5.2 (c), and HER-2 expression was scored as 2+ (d). Original magnification ×200.

E‑EMPD is a malignant neoplasm arising outside the common sites of EMPD and is exceedingly rare, accounting for less than 3% of all EMPD cases.^1^ Scarbrough et al. reported 45 E‑EMPD cases, with a predominance in Asians (29 Asians, 5 Whites, 11 unknown).^2^ Development of E-EMPD was associated with accessory mammary glands, which might arise on the milk line. Our case, consistent with previous reports, developed along the milk line, which represents the anatomical axis connecting the axilla, breast, and mons pubis. In a summary of 69 cases of E‑EMPD, Shimamura et al. reported that many lesions developed along the milk line.^1^

In the review of 45 E-EMPD cases, only one patient had lymph node metastasis and none had distant metastasis, suggesting a favorable prognosis.^2^ In contrast, our patient presented with advanced disease, likely due to prolonged neglect of the cutaneous lesion. In addition, HER‑2 immunostaining in our case showed a score of 2+. Prior studies have reported HER‑2 scores of 2+ or 3+ in 15%–32% of EMPD, correlating with dermal invasion and postoperative recurrence.^3^, ^4^ HER‑2 expression may therefore signify aggressive biological behavior in EMPD.

This case illustrates a rare clinical presentation mimicking Bowen’s disease, which was, however, excluded in that Paget cells exhibit clear cytoplasm and immunoreactivity for CEA, CAM5.2, and GCDFP‑15, whereas Bowen disease lacks these markers.^5^ Patches or plaques along the milk line that are clinically suspected to be Bowen’s disease should also be considered as possible E-EMPD. HER‑2 expression may contribute to disease progression and warrants further investigation as a prognostic and therapeutic target.

## Authors' contributions

Tatsuhiko Mori: Approval of the final version of the manuscript; data collection, analysis and interpretation; intellectual participation in propaedeutic and/or therapeutic management of studied cases; manuscript critical review; preparation and writing of the manuscript; study conception and planning.

Takehiro Nakamura: Data collection, analysis and interpretation; intellectual participation in propaedeutic and/or therapeutic management of studied cases.

Riko Ishizaki: Data collection, analysis and interpretation; intellectual participation in propaedeutic and/or therapeutic management of studied cases.

Kinuko Irie: Data collection, analysis and interpretation; intellectual participation in propaedeutic and/or therapeutic management of studied cases.

Takashi Ito: Data collection, analysis and interpretation; intellectual participation in propaedeutic and/or therapeutic management of studied cases.

Toshiyuki Yamamoto: Approval of the final version of the manuscript; intellectual participation in propaedeutic and/or therapeutic management of studied cases; manuscript critical review.

## Declaration of Generative AI and AI-assisted technologies in the writing process

During the preparation of this work, the authors used Microsoft Copilot in order to assist with language editing. After using this tool/service, the authors reviewed and edited the content as needed and take full responsibility for the content of the published article.

## Financial support

None declared.

## Research data availability

Does not apply.

## Conflicts of interest

None declared.
